# Identification of Potential Key Biomarkers and Immune Infiltration in Oral Lichen Planus

**DOI:** 10.1155/2022/7386895

**Published:** 2022-02-26

**Authors:** Lou Geng, Xingming Zhang, Yi Tang, Wenli Gu

**Affiliations:** Department of Clinical Laboratory, Shanghai Ninth People's Hospital, Shanghai Jiao Tong University School of Medicine, Shanghai 200011, China

## Abstract

**Background:**

Oral lichen planus (OLP) is a chronic autoimmune oral mucosal disease that seriously affects the life quality of the patients. But till now, the exact etiology and pathogenesis of OLP remain unclear. Our study is aimed at finding the key molecules and pathways involved in the pathogenesis mechanisms of OLP, providing more effective therapeutic strategies for OLP.

**Methods:**

Data from GSE52130 were downloaded from GEO datasets for analysis. Then, we carried out enrichment analysis of the differentially expressed genes (DEGs) using Gene Ontology (GO) and KEGG pathway analyses. Next, the CIBERSORT algorithm was used to assess immune cell infiltration in OLP patients. Furthermore, we also constructed a protein-protein interaction network using STRING and Cytoscape and simultaneously sought potential transcription factors plug-in including MCODE CytoHubba and iRegulon. In addition, ROC analysis was employed to assess the diagnostic performance of these hub genes. Lastly, we identified 6 promising novel drugs to treat OLP through Connectivity Map.

**Results:**

We illustrated that 255 DEGs were mainly enriched in the focal adhesion pathway and metabolism pathways. Besides, Cibersort analysis showed that M1 macrophages, T follicular helper cells, and T regulatory cells are more infiltrated in OLP samples. In addition, ROC analysis demonstrated that these hub genes owned higher diagnostic value in OLP, in which SPRR1B had the highest diagnostic value. And we also predicted that SOX7 was the most relevant transcription factor of those hub genes. Lastly, through the CMap database, we identified 6 small molecules as possible treatment drugs of OLP.

**Conclusion:**

Our research identified that SPRR1B could be used as potential biomarkers for the early diagnosis of OLP. In addition, as a chronic autoimmune oral mucosal disease, OLP has different infiltration types of immune cells. Furthermore, 6 small molecules were proposed as promising novel treatment drugs for OLP patients. Therefore, our research may provide new impetus for the development of effective OLP biological treatment options.

## 1. Introduction

Oral lichen planus (OLP) is a chronic autoimmune oral mucosal disease affecting 1-2% population worldwide [1, 2] and is characterized by its recurrence and chronic protraction course. It can be divided into erosive and nonerosive OLP according to the condition of the base mucosa of the lesion. The disease mainly invades buccal mucosa, tongue, and gingiva, so most patients may manifest ulcers, erosion, papules, and mucosal exfoliation in the oral cavity, while others obtain rough, numb, and painful feelings. Pain is one of the OLP common symptoms and may even interfere with the patient's speech, eating, and swallowing.

It has been known that infiltration of T lymphocytes in lamina propria, formation of keratinocytes, and destruction of basement membrane is typical histopathological features of OLP. The helper T cells are the main lymphocytes in the OLP, which can activate cytotoxic T cells and aggravate the local immune response and thus induce apoptosis of keratinocytes [3].

But till now, the exact etiology and pathogenesis of OLP remain unclear. At present, it is recognized that many pathogenic factors, such as autoimmune response, mental stress, infection, and hypersensitivity, might be involved in the development of OLP [4]. The treatment of this disease is mainly with glucocorticoids, nystatin, surface anesthesia, antibiotic mouthwash, and so on, but the effect is not ideal. Meanwhile, these local or systemic immunosuppressors can usually only alleviate clinical symptoms and have obvious side effects for long-term use [5]. Therefore, elucidating the mechanisms underlying the pathogenesis and identifying more effective therapeutic strategies for OLP is essential.

Moreover, OLP has been regarded as one of the precancerous lesions of oral squamous cell carcinoma (OSCC), which is the most dominant type of oral cancer, accounting for more than 90% of them [6]. As a result, it is urgent to clarify the malignant transformation mechanism of OLP to OSCC, which might benefit to improve the ability of early diagnosis of OSCC and provide new and more effective treatment measures.

## 2. Materials and Methods

### 2.1. Acquisition of Sample Information

Firstly, we got the clinical sample's information of OLP from the Gene Expression Omnibus (GEO) database (https://www.ncbi.nlm.nih.gov/geo/). 23 tissue samples were retrieved from GSE52130; we selected the top 14 ones, including 7 OLP epithelium samples (OLPE) and 7 healthy control oral epithelium (COE) samples. OLP patients were diagnosed clinically and histologically according to WHO diagnostic criteria.

To do further analysis, we downloaded all the mRNA information of these 14 selected samples.

### 2.2. Data Further Process

Next, we normalized and processed the original expression matrix using R. After that, we also selected out differentially expressed genes (DEGs) by the limma package [7], based on the criteria: at least a 1.5-fold change between COE samples and OLPE samples and with adjusted *P* value < 0.05.

### 2.3. Enrichment Analysis

Next, all genetic information of OLPE and COE samples was uploaded to gene set analysis (GSEA); in addition, we also uploaded the 255 differentially expressed genes to Ingenuity Pathway Analysis (IPA) database to perform canonical pathway and molecule function analysis. The default parameters were as follows: both *P* value < 0.05 and absolute value of *z*-score > 2 are considered significant.

### 2.4. Gene Ontology (GO) and Pathway Enrichment Analyses

We next perform GO and KEGG enrichment analyses of the DEGs. GO includes three categories, respectively, molecular function (MF), biological processes (BP), and cellular components (CC) [8]. KEGG is a knowledge database for systematic analysis of gene function in terms of the networks of genes [9]. On the other hand, Reactome is a pathway database which provides intuitive bioinformatics tools for the visualization, interpretation, and analysis of pathway knowledge. In this part of the study, *P* < 0.05 was used as the threshold value, and the number of genes enriched in each pathway was ≥2.

### 2.5. Immune Infiltration Analysis

The CIBERSORT algorithm was used to estimate the relative abundance of 22 human immune cell types for purpose of elucidating the immune infiltration landscape of OLP. It could evaluate the infiltration proportions of immunocyte types in OLP samples by using the LM22 gene signature based on deconvolution [10]. We loaded DEGs between normal and OLP tissue samples into the CIBERSORT website (https://ciberfortstanford.edu/), and the threshold value was set to a *P* value < 0.05. Then, we obtained the immune score of 22 immune cells and visualized them by using R packages “ggplot2.”

### 2.6. Gene Cluster Identification and Protein-Protein Interaction (PPI) Network Analysis

The DEGs in OLPE samples were uploaded to STRING to obtain the protein network interaction diagram, in which a combination score of >0.4 was set as a threshold value [11]. Next, the result of STRING analysis was imported into Cytoscape v.3.7.2, and protein cluster analysis of differential expressed genes was conducted using Molecular Complex Detection (MCODE) plug-in [12]. The genes contained in the gene cluster with the highest scores were imported into the STRING to draw the protein interaction network and further analysis of which biological processes this gene cluster was participated in. Accordingly, we could regard the nodes with higher degrees of interaction as hub nodes.

### 2.7. ROC Analysis

The GSE38616 dataset was downloaded from the GEO database and used as validation datasets. Receiver operating characteristic (ROC) curve analysis was used to evaluate the diagnostic value of these hub genes. *P* value < 0.05 was considered statistically significant.

### 2.8. Analysis of Hub Genes and Transcription Factors Associated with OLP

CytoHubba is a Cytoscape plugin app, which has provided a simple and convenient method to explore key nodes in biological networks and select the degree method to probe the PPI network for hub genes.

Subsequently, the Cytoscape plugin iRegulon was used to analyze transcription factors regulating marker genes [13]. Parameter settings were as follows: minimum identity between orthologous genes = 0.05 and maximum false discovery rate on motif similarity = 0.001. The normalized enrichment score (NES) was the output result. The higher the scores were, the more reliable the results were. Transcription factors and target gene pairs with NES > 7 were selected.

### 2.9. CMap Analysis

The Connectivity Map (CMap) (https://portals.broadinstitute.org/cmap) is an effective tool for predicting potential drugs that may affect the biological state encoded in gene expression signatures [14]. The enrichment score indicative of similarity was calculated, ranging from −1 to 1. A positive connectivity score indicated that the drug could induce a similar pathway of disease progression, whereas a negative one revealed that the drug could be a therapeutic drug for OLP.

## 3. Results

### 3.1. Sample Information Processing and Screening of Differentially Expressed Genes

**Figure 1:**
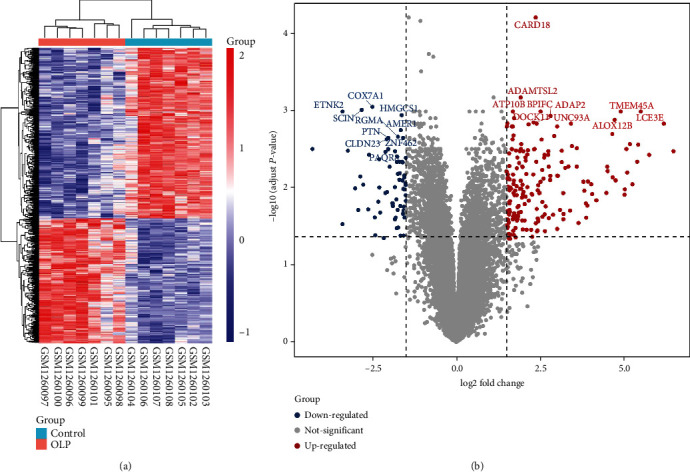
The differentially expressed genes between OLPE and COE were shown on the heat map and the volcano plot. (a) Heat map of differentially expressed genes. Blue color represented a lower, and red color represented a higher expression level, while grey color demonstrated no differential expression. (b) The volcano plot of differentially expressed genes. Blue dots represented significantly downregulated genes, while red dots represented significantly upregulated genes. COE: control oral epithelium; OLPE: oral lichen planus epithelium samples.

Based on the sample information, 255 differentially expressed genes (DEGs) picked out from the OLPE samples, in which 187 genes were upregulated and 68 genes were downregulated. The screening criteria for DEGs were as follows: adjust *P* value < 0.05 and ∣log_2_ Fold Change | ≥1.5. According to the analysis of these gene expressions, the heat map and the volcano plot are made as shown in Figure 1.

### 3.2. GO and KEGG Enrichment Analyses

**Figure 2:**
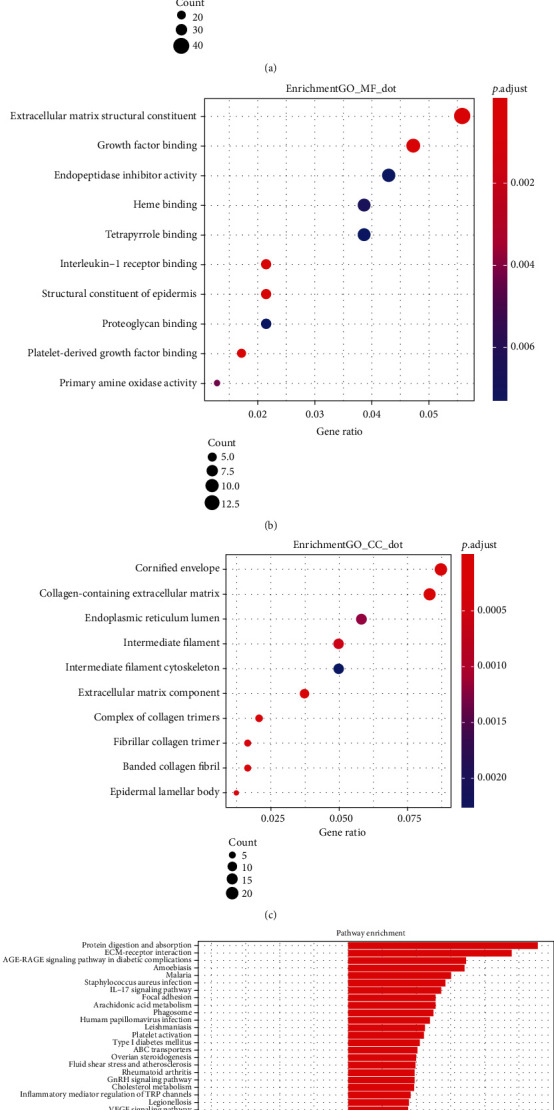
GO and KEGG enrichment analyses were conducted on 255 DEGs in the OLPE samples. (a) Biology process enrichment of the DEGs. (b) Molecular function enrichment of the DEGs. (c) Cellular component enrichment of the DEGs. (d) KEGG pathway enrichment of the DEGs.

Then, GO and KEGG enrichment analyses were conducted on 255 DEGs in the OLPE samples by using R. Genes ontology and biological functional analysis indicated that the 255 DEGs in OLPE samples were most related to skin development in the biological process, and extracellular matrix structural constituent in the molecular function, in addition, cornified envelope in the cellular component as shown in Figures 2(a)–2(c).

On the other hand, KEGG pathway analysis showed the 255 DEGs genes were significantly enriched in protein digestion and absorption, ECM-receptor interaction, focal adhesion, and metabolism pathways, such as drug metabolism-cytochrome P450, glycine, serine and threonine metabolism, and tryptophan metabolism, as shown in Figure 2(d).

In addition, analysis of the pathway enrichment indicated that DEGs in OLPE samples were mainly enriched in the IL-17 signaling pathway. It has been well known that OLP is an autoimmune disease, in which IL-17 mRNA and protein with higher expression, so the IL-17 signaling pathway may have a critical role in OLP. And recent research found that Renin could significantly upregulate the expression of IL-17 by promoting STAT4 phosphorylation in oral keratinocytes, which provided promising potential targeted therapies for OLP patients [15].

### 3.3. The DEGs in the OLPE Samples Were Mainly Enriched in Focal Adhesion Pathway and Metabolism Pathway

**Figure 3:**
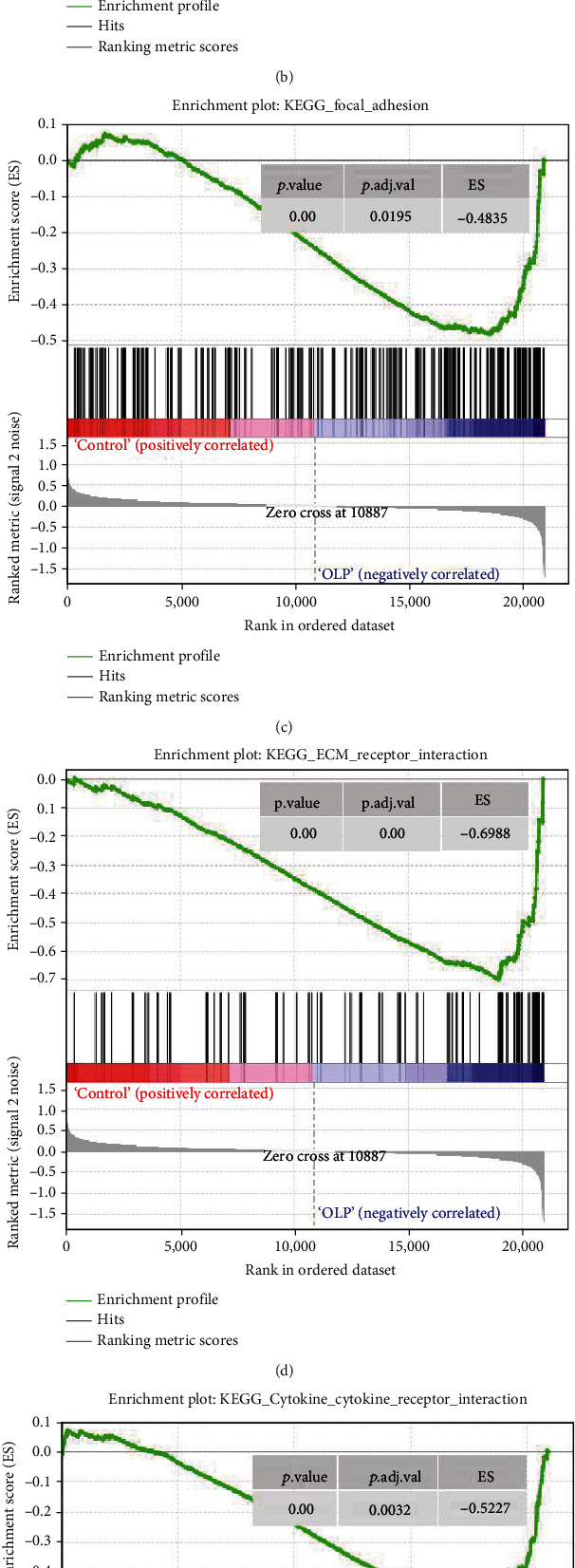
h.all. v 6.2.symbols.gmt (Hallmarks) gene set database was used to analyze the whole gene expression value of the OLPE and COE samples. GSEA first filtered the gene set according to the number of genes contained in the gene set, with the minimum number of 15 genes and the maximum number of 500 genes by default. Significant gene sets were cut-off by FDR < 0.25 and *P* value < 0.05.

Gene set enrichment analysis (GSEA) was used for enrichment analysis of the samples' genes. The significantly enriched gene sets were set at a default cut-off as *P* value < 0.05 and FDR < 0.25. The enrichment analysis of gene sets indicated that the focal adhesion pathway, ECM-receptor interaction, primary immunodeficiency, autoimmune thyroid disease, chemokine signaling pathway, and cytokine receptor interaction were significantly enriched in OLPE samples as shown in Figure 3. Accordingly, it was reasonably concluded that OLP might be related to primary immunodeficiency diseases and autoimmune driven diseases, such as autoimmune thyroid disease. Moreover, the enrichment analysis also showed that there are some common pathways and pathophysiological mechanisms between OLP and immune diseases, which gives us a deeper understanding of OLP.

In addition, it was shown that the KEGG and Reactome pathways of upregulated DEGs were significantly enriched in ECM-receptor interaction, protein digestion, absorption, amoebiasis, and focal adhesion, but those pathways of downregulated DEGs were enriched in biological oxidations, drug metabolism-cytochrome P450, and metabolic pathways, as shown in Table 1.

### 3.4. Further Pathway Analysis by Using IPA

**Figure 4:**
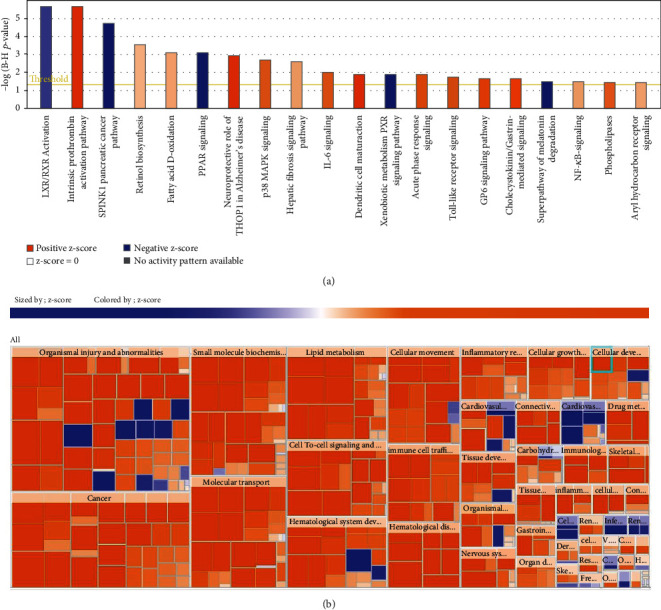
Further pathway analysis by using IPA was conducted on 255 DEGs in the OLPE samples. (a) The canonical pathway analysis of IPA. The color depth in the bar chart represented the *z*-score, and generally, an absolute *z*-score greater than 2 was considered meaningful. (b) Disease and function analysis of IPA. Orange meant *z* − score > 0, blue meant *z* − score < 0, and grey meant no *z*-score; *z* − score > 2 meant the function was significantly activated, and *z* − score < −2 means the function was significantly inhibited.

To further validate our results and identify crucial molecules involved in the progress of the OLP, a total of 255 differentially expressed genes were uploaded to Ingenuity Pathway Analysis (IPA) for core analysis. We used the IPA software to further validate the results of DEGs. A total of 20 canonical pathway analysis results showed that 9 pathways were activated and 4 pathways were inhibited significantly, as shown in Figure 4(a) and Table 2. Specifically, intrinsic prothrombin activation pathway had the highest activation scores.

On the other hand, the heat map could indicate the results of disease and function analysis, which showed there were 10 main functional modules about 255 differentially expressed genes in the OLPE samples, namely, organismal injury and abnormalities, renal and urological disease, cancer, small molecule biochemistry, molecular transport, lipid metabolism, cell-to-cell signaling and interaction, hematological system development and function, cellular movement, hematological system development and function, and immune cell trafficking.  Figure 4(b) and Table 3 show significantly activated and inhibited functions among these main modules. From the point of view of the analysis, we could draw conclusions that OLP might have a close relationship with tumor, and those upregulated DEGs were mainly enriched in cell migration and movement pathways. In addition, the analysis results also indicated that OLP's occurrence might be related to hepatitis C virus infection.

### 3.5. Analysis of Immune Cell Infiltration

**Figure 5:**
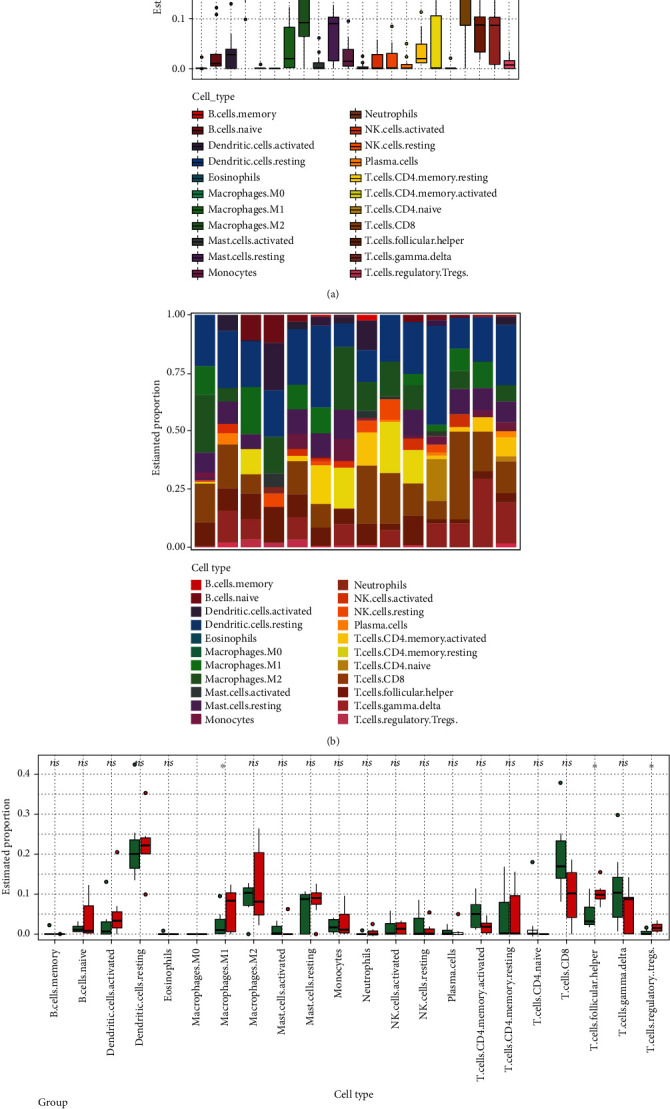
The abundance of 22 infiltrating immune cell types in OLP was showed. (a) Barplot showed the proportion of 22 types of infiltrating immune cell in OLP samples. (b) Heat map showed the abundance of immune cells in each sample. (c) Barplot showed the proportion of each immune cell type between normal and OLP samples, in which the green represented normal samples and the red represented OLP samples. *P* value < 0.05.

As OLP was an immune-driven disease and we also identified some immune-related pathways including the IL-17 signaling pathway, dendritic cell maturation, chemokine signaling pathway, and cytokine receptor interaction, so we used the CIBERSORT algorithm to estimate the abundance of immune cells in OLP. The results revealed that macrophages M1, activated dendritic cells, T cell follicular cells, and CD8 T cells account for a large proportion of immune cells, as shown in Figure 5(a). The distribution of 22 types of immune cells in each sample showed the immunological differences between OLP samples and control ones, as shown in Figure 5(b). And the box plot in Figure 5(c) visualizes those differences in each type of immune cell. The results showed that OLP samples displayed a significantly increased abundance of T cell regulatory (Tregs), macrophages M1, and T cell follicular helper than the control.

### 3.6. Protein-Protein Interaction (PPI) Network Analysis

**Figure 6:**
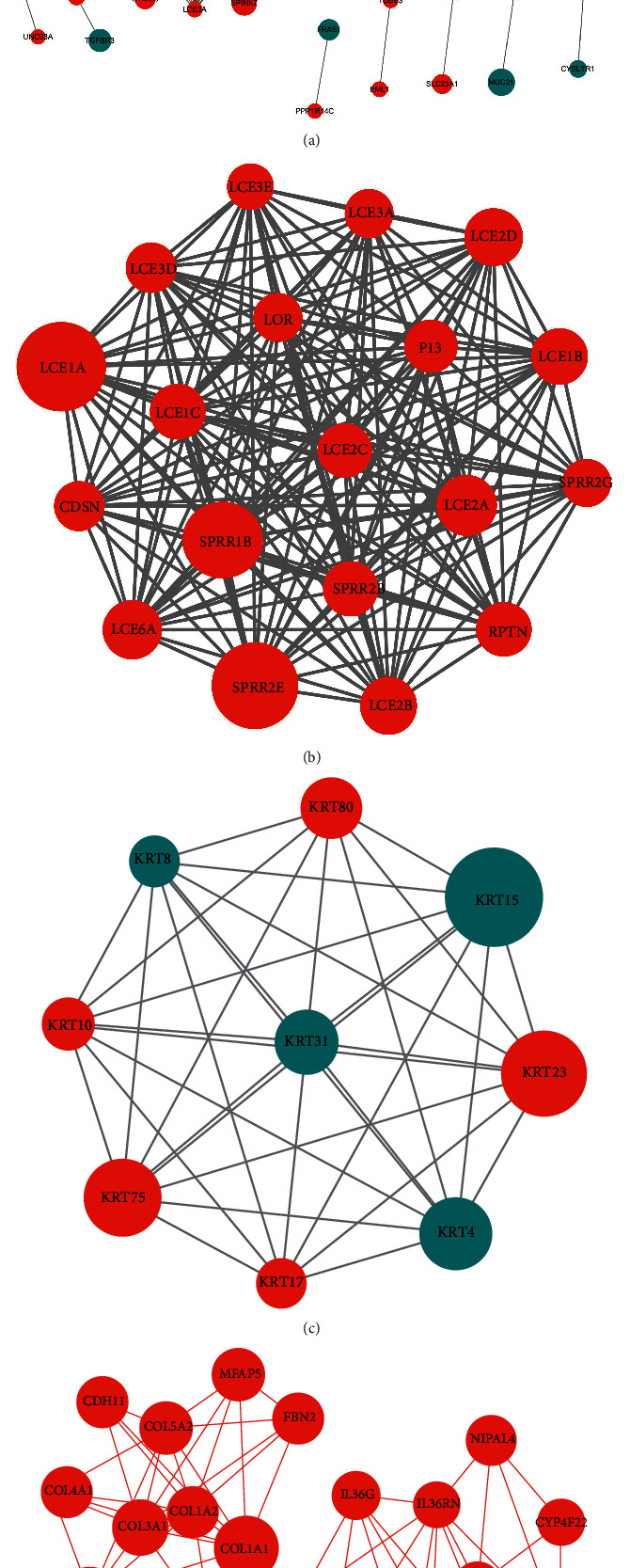
Protein-protein interaction network analysis was processed with Cytoscape, and different clusters were analyzed by MCODE. (a) The STRING database file was uploaded into Cytoscape v.3.7.2 to draw protein-protein interaction network, and different clusters analyzed by MCODE were noted with different colors. The significance of *P* value was shown by the size of node. The color of the nodes showed the change of the DEGs, and the smaller the *P* value is, the larger the diameter of node is. The color of the edge represented the value of combined score from 0.4 to 1, grey to dark. (b). The cluster 1 had 19 nodes and 171 edges, and the cluster score is 19.00. (c) The cluster 2 network had 9 nodes and 36 edges with 9.00 cluster score. (d) The cluster 3 network had 29 nodes and 89 edges with 6.357 cluster score, in which the upregulated nodes were colored in red, but the downregulated ones were colored in blue.

To filter out the hub genes from the differentially expressed genes in the OLPE sample, we next uploaded 255 differentially expressed genes to the STRING for further analysis and obtained 245 nodes and 586 edges. The local clustering coefficient was 0.411 and PPI enrichment *P* value < 1.0*e* − 16; then, the TSV format file was downloaded and processed with Cytoscape as shown in Figure 6(a). MCODE was used to process the network data to identify gene clusters as shown in Figures 6(b)–6(d), and genes in the first three-gene cluster with the highest score ranking were selected for BP enrichment analysis. Next, we imported these cluster genes into the Metascape database to analyze and then found that the genes in the gene cluster 1 and cluster 2 were mainly involved in cornification and formation of the cornified envelope as shown in Table 4, which was consistent with the pathological results and basic pathological process of oral lichen planus.

But actually, what interested us most was cluster 3, which enriched more related ways and might play more important roles in OLP. The 29 genes in gene cluster 3 mainly participated in posttranslational protein phosphorylation, inflammatory response to antigenic stimulus, fever generation, blood vessel development, signaling by PDGF, response to nutrient levels, response to inorganic substance, negative regulation of cytokine-mediated signaling pathway, and regulated exocytosis pathway, all of which were with high statistical significance according to *P* values.

### 3.7. Hub Genes and Transcription Factors Associated with OLP

**Figure 7:**
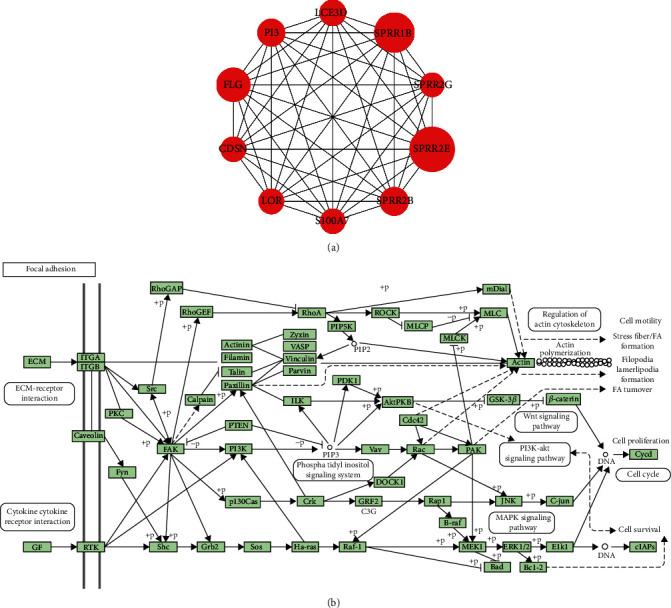
Hub genes associated with OLP were showed on the hub gene network and the focal adhesion diagram. Focal adhesion KEGG pathway diagram. (a) Top 10 genes were identified as hub genes by means of degree method, all of which were upregulated. (b) The focal adhesion pathway diagram was downloaded from KEGG website.

Using the degree method, we identified LOR, CDSN, PI3, FLG, LCE3D, S100A7, SPRR1B, SPRR2G, SPRR2B, and SPRR2E as hub genes, as shown in Figure 7(a). Furthermore, we downloaded the focal adhesion KEGG pathway diagram, as shown in Figure 7(b). Focal adhesion and PI3K-Akt signaling pathway, ECM-receptor interaction, and cytokine-cytokine receptor interaction exit a crosslink. It was well known that the focal adhesion pathway plays a major role in the pathogenesis of OLP, which was also related to hematopoiesis, tumor metastasis, vascular diseases, and malignant transformation. On the other hand, the metabolism disorder had a close relationship with the development of OLP. It had been shown that the HIF1*α*/PLD2 axis was associated with glycolysis and induces T cell immunity in oral lichen planus [16]. As metabolic changes are significant during the malignant transformation of primary OLP cells, it is important to focus on changes in metabolism.

**Figure 8:**
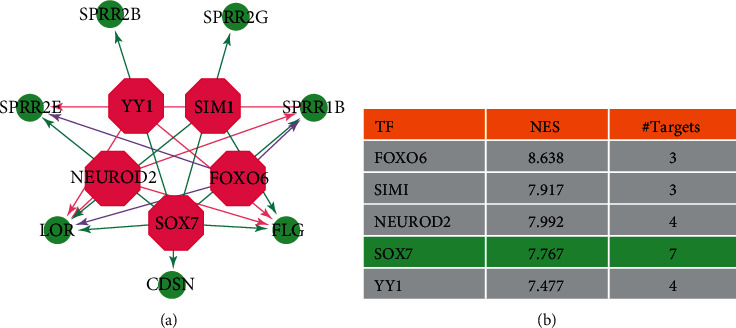
We showed the transcription regulatory network of these hub genes. (a) Five transcription factors with an NES score > 7 were predicted by iRegulon and visualized by the Cytoscape. We showed regulatory network between transcription factors and targeted genes, in which SOX7 could overlap with seven genes, so it might play a more important role in the progression of OLP. (b) The normalized enrichment score (NES) of SOX7 was 7.767.

In addition, the transcription regulatory network of these hub genes was shown in Figure 8. Among them, the transcription factors with an NES score > 7 consisted of FOXO6 (Forkhead Box O6, NES = 8.638), SIM1 (SIM BHLH Transcription Factor 1, NES = 7.917), NEUROD2 (Neuronal Differentiation 2, NES = 7.992), SOX7 (SRY-Box Transcription Factor 7, NES = 7.767), and YY1 (YY1 Transcription Factor, NES = 7.477). Since SOX7 targets more hub genes, it plays a more important role in the progression of OLP.

### 3.8. Construction and Validation of the Prognostic Model

**Figure 9:**
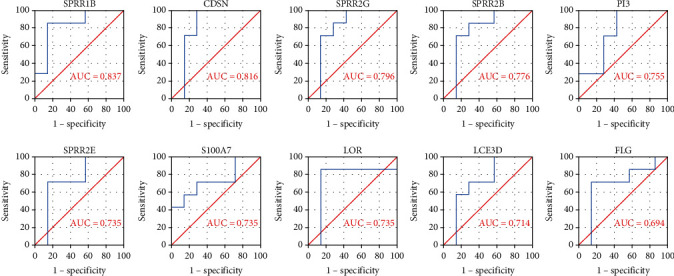
ROC curve of the 10 specifically expressed hub genes for OLP. We conducted ROC analysis to evaluate diagnostic performance of 10 specifically expressed hub genes and used area under the curve (AUC) to indicate the intrinsic effectiveness of diagnostic tests. It was showed that SPRR1B had the highest diagnostic value (AUC: 0.837), and other genes AUC were in turn as follows: CDSN (AUC: 0.816), SPRR2G (AUC: 0.796), SPRR2B (AUC: 0.776), PI3 (AUC: 0.755), SPRR2E (AUC: 0.735), S100A7 (AUC: 0.735), LOR (AUC: 0.735), LCE3D (AUC: 0.714), and FLG (AUC: 0.694). Therefore, we hypothesized that SPRR1B might be biomarkers for early diagnosis of OLP.

Dataset GSE38616 was treated as the validation set; we conducted ROC analysis to evaluate the diagnostic performance of 10 specifically expressed hub genes and used area under the curve (AUC) as an indicator combining sensitivity and specificity, which could describe the intrinsic effectiveness of diagnostic tests. It was shown that those hub genes owned higher diagnostic value in OLP, in which SPRR1B had the highest diagnostic value (AUC: 0.837). And the AUC of other genes in OLP were in turn as follows: CDSN (AUC: 0.816), SPRR2G (AUC: 0.796), SPRR2B (AUC: 0.776), PI3 (AUC: 0.755), SPRR2E (AUC: 0.735), S100A7 (AUC: 0.735), LOR (AUC: 0.735), LCE3D (AUC: 0.714), and FLG (AUC: 0.694), as shown in Figure 9. Therefore, we hypothesized that SPRR1B might be biomarkers for the early diagnosis of OLP.

### 3.9. Identification of Potential Compounds

To identify the potential drugs for regulating the progression of OLP, we applied the upregulated and downregulated tags to query the CMap database. As presented in Table 5, the top positively correlated compounds included pimethixene, caffeic acid, proadifen, clenbuterol, withaferin A, cinnarizine, molindone, and parthenolide, which might make disease deterioration. On the contrary, the top 6 negatively correlated compounds had a relatively prominent function in reversing differential expression during OLP progression, and they were AG-013608, Prestwick-857, harmalol, bumetanide, MK-886, and NU-1025. These findings suggested that these 6 small molecule compounds might be potential drugs for OLP patients. However, the above conclusions need to be further verified.

## 4. Discussion

OLP is a common chronic mucocutaneous inflammatory disease and is also regarded as a potentially malignant oral disorder by WHO because 1.63% of lesions initially diagnosed as OLP evolved into OSCC. In this study, we screened 255 differentially expressed genes from OLP epithelium samples through the array dataset GSE52130, in which 187 upregulated and 68 downregulated genes. Next, the results of gene enrichment analysis suggested that the differentially expressed genes in the OLPE samples were mainly enriched in the focal adhesion pathway and metabolism pathway.

It has been known that metabolism become the focus of the etiology of immune diseases, which could coordinate the proliferation and differentiation of T cells [17]. And OLP is T cell-mediated inflammatory disorder, and metabolic pathway has been regarded as an important part of the OLP pathophysiological mechanism. Furthermore, the metabolic changes of OLP cells are closely related to the development of disease and malignant transformation. Studies have shown that succinate accumulates in OLP and OSCC at both tissue and cell levels, which activates the hypoxia-inducible factor-1*α* (HIF-1*α*) pathway and induces apoptosis, so succinate plays a key role in metabolic changes during the malignant transformation from OLP to OSCC [17].

Other researchers found that the mTOR pathway was upregulated in OLP patients, which played an important role in the immune metabolism of T cells. Targeted mTOR glycolysis pathway could significantly inhibit the proliferation of T cells and block its DNA synthesis, thereby inducing cell apoptosis and regulating Th17 subsets differentiation [18]. In addition, HIF1*α* and phospholipase D2 (PLD2) are highly expressed in local T cells of OLP, and HIF1*α* could upregulate the expression of PLD2 and promote T cellular immunity of OLP through glycolysis [16]. So, the metabolic changes of OLP are especially worthy of our further exploration, and the intervention to OLP metabolism might be a new therapeutic schedule.

On the other hand, the adhesion pathway is enriched in various diseases including cancer. As we known, the focal adhesion and ECM-receptor interaction pathways have been found to be involved in the development of OSCC [19]. In addition, it had been reported that HPV-mediated cervical malignancy might disrupt the process of cell homeostasis because of local immunosuppression, then damage the focal adhesion and decomposition of extracellular matrix, therefore promote the invasion, diffusion, and metastasis of cancer cells [20]. Besides, it has been found that integrin *α*3 could recruit the c-Src/extracellular signal-regulated protein kinase cascade and induce the phosphorylation of focal adhesion kinase, thus enhance migration and invasion of cervical cancer cells and promote angiogenesis through matrix metalloproteinase 9 [21]. Therefore, targeting the focal adhesion pathway is expected to become a new therapeutic strategy to slow down the development of the disease and improve the patient's condition.

As the enrichment analyses of OLP have showed its correlation with immune function, we then conducted Cibersort analysis. The immune infiltration profiles were different between normal and OLP samples, and the OLP displayed increased abundance of Tregs, macrophages M1, and T cell follicular helper. It has been well known that Tregs have emerged as important mediators in inflammatory and autoimmune diseases. Researchers had found that OLP patients had a higher proportion of Tregs both in serum and in tissues than healthy ones, which suggested that Tregs might contribute to the immunopathogenesis of OLP, and it might provide a new therapeutic target for OLP treatment [22]. As reported, macrophages could involve in the immunopathogenesis of OLP; in addition, CD68^+^ macrophages could serve as a diagnostic indicator of OLP [23]. Furthermore, the pathogenetic functions of T follicular helper cells, a subtype of CD4+ T-helper cells, significantly increased in OLP, which also were involved in pathogenesis of OLP [22]. Therefore, our findings might be essential to future target studies of OLP immunotherapies.

Filaggrin (FLG) and loricrin (LOR) are skin barrier protein, and LOR is a major cell envelopes (CE) component. It had been reported that LOR and FLG were abnormally upregulated in atopic dermatitis, which played a critical role in disease development [24]. It has been well known that OLP is a hyperkeratotic mucosal disease, and recently, researchers have found that the expression of filaggrin and filaggrin-2 markedly increased in OLP patients [25], which might suggest that filaggrin is essential to keratinization. Our findings about FLG also provide strong evidence for this viewpoint. In addition, late cornified envelope protein 3D (LCE3D), one of hub genes we have found, is also a specific development associated gene, which participated in the formation of stratum corneum, and was associated with psoriasis vulgaris and atopic dermatitis [26].

Corneodesmosin (CDSN) is identified as an adhesive protein maintaining cohesion and intercellular integrity in skin. And the deficiency of CDSN in mouse skin causes epidermal barrier defects including severe skin detachment, increased transepidermal water loss, and easy penetration of toluidine blue [27]. But until now, the function of CDSN in OLP has not been reported.

PI3 is also known as elafin, an endogenous serine protease inhibitor produced by epithelial and immune cells. Research has shown that elafin is closely related to innate immune, and proteolytic cleavage of elafin may impair the innate immune function of the protein [28]. In addition, elafin expression levels and subcellular localization could be used as a biomarker for cervical cancer severity [28]. In our study, we also found that PI3 mRNA expression was significantly upregulated in OLP patients. From our knowledge, it is the first report that PI3 might be acted as a novel biomarker in OLP.

S100A7, s100 calcium binding protein A7, is a member of S100 protein family. Researchers have found that it was abnormally upregulated in esophageal squamous cell carcinoma (ESCC), which might promote tumor progression by impacting M2 macrophage infiltration and angiogenesis; thereby, S100A7 was expected to act as a therapeutic target for ESCC treatment [25]. Moreover, based on whole gene expression profiling, it had been identified as a novel candidate biomarker related to OSCC [29].

It has been well known that SPRR family molecules played important functions in the progression of many diseases, so SPRR families, especially SPRR1B, may be potential predictive biomarkers of lung adenocarcinoma [30]. Research has found that overexpression of SPRR1B in OSCC stem-like cells was positively correlated with these cells growth by activating of MAP kinase signal [30]. In addition, overexpression of SPRR2B could promote cell proliferation in gastric adenocarcinoma by MDM2-p53/p21 signal pathway [31]. Moreover, researchers also found that SPRR2E was one of gene prognostic signature for OSCC [32]. In our study, we have identified that SPRR1B might be acted as potential predictive biomarkers and treatment targets in OLP.

Furthermore, we also identified that FOXO6, SIM1, NEUROD2, SOX7, and YY1 might be the regulators of OLP, especially SOX7, which would be worthy of further exploration in order to provide clues for improving clinical treatment effects of OLP.

Currently, there are no effective treatments for OLP, due to its unknown etiology. In this respect, based on the analysis of DEGs, we also selected 6 drugs which might help to develop new targeted drugs for OLP by using the CMap database. NU-1025 is a PARP inhibitor, which could sensitize temozolomide-treated glioblastoma cell lines and decrease drug resistance [33]. Besides, NU-1025 can inhibit cell proliferation and induce apoptosis in human breast cancer cells [34].

MK-886, an inhibitor of the 5-lipoxygenase-activating protein (FLAP), could suppress leukotriene biosynthesis and reduce choroidal neovascularization in age-related macular degeneration models [35]. Literature reviews show that bumetanide has a promising effect in many diseases, including organ fibrosis, neonatal epilepsy, and heart failure [36]. Harmalol, a beta carboline alkaloid, can induce apoptosis in HepG2 via binding to DNA sequences [37]. Besides, AG-013608 and Prestwick-857 have not yet been reported as an active drugs for any disease. Therefore, our research may provide new impetus for the development of effective OLP biological treatment options.

## 5. Conclusion

Our research demonstrated that SPRR1B could serve as potential biomarkers for the early diagnosis of OLP and identified 6 small molecules as promising novel treatment drugs for OLP patients.

## Figures and Tables

**Table 1:** KEGG and Reactome pathway enrichment analyses for differentially expressed genes (*P* < 0.05).

ID	Terms	Count	*P* value
Upregulated			
hsa04512	ECM-receptor interaction	9	3.06*E*-07
hsa04974	Protein digestion and absorption	8	4.79*E*-06
hsa05146	Amoebiasis	7	1.65*E*-04
hsa04510	Focal adhesion	9	1.80*E*-04
hsa04611	Platelet activation	6	0.00336272
hsa04151	PI3K-Akt signaling pathway	9	0.005079306
hsa04145	Phagosome	6	0.006175871
hsa05144	Malaria	4	0.006529778
hsa04726	Serotonergic synapse	5	0.0109323
hsa00590	Arachidonic acid metabolism	4	0.011940849
hsa05323	Rheumatoid arthritis	4	0.031412058
hsa04912	GnRH signaling pathway	4	0.034212174
hsa04750	Inflammatory mediator regulation of TRP channels	4	0.041234535
hsa04940	Type I diabetes mellitus	3	0.042639686
hsa02010	ABC transporters	3	0.046388375
Downregulated			
R-HSA-211859	Biological oxidations	11	1.950*E*-7
R-HSA-5579029	Metabolic disorders of biological oxidation enzymes	3	1.380*E*-4
hsa00982	Drug metabolism-cytochrome P450	4	0.002280447
hsa00340	Histidine metabolism	3	0.003265313
hsa00260	Glycine, serine, and threonine metabolism	3	0.010053474
hsa00380	Tryptophan metabolism	3	0.010557146
hsa00330	Arginine and proline metabolism	3	0.016186014
hsa01100	Metabolic pathways	10	0.037458967

**Table 2:** IPA canonical pathways.

Pathways	-log *P*	*Z*-score
Activation pathways		
Intrinsic prothrombin activation	5.68	2.828
Neuroprotective role of THOP1 in Alzheimer's disease	2.9	2.828
Dendritic cell maturation	1.9	2.646
GP6 signaling pathway	1.63	2.449
Cholecystokinin/gastrin-mediated signaling	1.62	2.449
Acute phase response signaling	1.88	2.236
Toll-like receptor signaling	1.74	2.2361
p38 MAPK signaling	2.72	2.121
Phospholipases	1.42	2.000
Inhibitory pathways		
SPINK1 pancreatic cancer pathway	4.74	-2.828
PPAR signaling	3.07	-2.121
PXR signaling pathway	1.88	-2.121
Super pathway of melatonin degradation	1.47	-2.000

**Table 3:** IPA significant functions.

Functions	*Z*-score	*P* value
Activated functions		
Release of lipid	2.93	1.2*E*-03
Cancer	2.77	2.56*E*-05
Release of fatty acid	2.764	1.08*E*-03
Release of eicosanoid	2.592	1.48*E*-03
Growth of malignant tumor	2.436	1.98*E*-03
Quantity of catecholamine	2.376	1.76*E*-03
Release of prostaglandin E2	2.361	1.86*E*-6
Transport of molecule	2.265	7.78*E*-4
Cancer of cells	2.228	1.28*E*-6
Activation of phagocytes	2.211	8.66*E*-4
Concentration of dopamine	2.204	1.81*E*-3
Neoplasia of cells	2.190	3.32*E*-4
Leukocyte migration	2.150	1.51*E*-3
Cell movement of leukocytes	2.125	6.93*E*-4
Malignant solid tumor	2.093	1.49*E*-5
Cell movement of granulocytes	2.078	4.2*E*-3
Neurotransmission	2.049	5.08*E*-3
Activation of myeloid cells	2.003	1.19*E*-3
Inhibited functions		
Replication of hepatitis C virus	-2.219	3.74*E*-3

**Table 4:** Top 3 gene cluster analysis by Metascape database.

Cluster	Terms	Count	*P* value
Cluster 1			
GO:0070268	Cornification	9	-21.14
Cluster 2			
R-HSA-6809371	Formation of the cornified envelope	18	-40.17
GO:0070268	Cornification	9	-16.2
Cluster 3			
R-HSA-8957275	Posttranslational protein phosphorylation	6	-8.54
GO:0002437	Inflammatory response to antigenic stimulus	5	-7.92
GO:0001660	Fever generation	3	-6.74
GO:0001568	Blood vessel development	9	-6.74
R-HSA-186797	Signaling by PDGF	4	-6.18
GO:0031667	Response to nutrient levels	7	-5.8
GO:0010035	Response to inorganic substance	6	-4.34
GO:0001960	Negative regulation of cytokine-mediated signaling pathway	3	-4.05
GO:0045055	Regulated exocytosis	6	-3.53

**Table 5:** Small molecule drugs identified by Connectivity Map.

Drugs	Enrichment
Positive compounds	
Pimethixene	0.811
Caffeic acid	0.797
Proadifen	0.752
Clenbuterol	0.711
Withaferin A	0.708
Cinnarizine	0.649
Molindone	0.642
Parthenolide	0.633
Negative compounds	
AG-013608	-0.455
Prestwick-857	-0.719
Harmalol	-0.751
Bumetanide	-0.765
MK-886	-0.909
NU-1025	-0.935

## Data Availability

Publicly available datasets were analyzed in this study. This data can be found here: https://www.ncbi.nlm.nih.gov/geo/.
